# Approach to adult patients with primary hypothyroidism in some special situations: a position statement from the Thyroid Department of the Brazilian Society of Endocrinology and Metabolism (SBEM)

**DOI:** 10.20945/2359-3997000000545

**Published:** 2022-11-28

**Authors:** Gláucia Maria Ferreira da Silva Mazeto, José Augusto Sgarbi, Helton Estrela Ramos, Danilo Glauco Pereira Villagelin, Célia Regina Nogueira, Mario Vaisman, Hans Graf, Gisah Amaral de Carvalho

**Affiliations:** 1 Universidade Estadual Paulista Faculdade de Medicina de Botucatu Departamento de Clínica Médica Botucatu SP Brasil Departamento de Clínica Médica, Faculdade de Medicina de Botucatu, Universidade Estadual Paulista (Unesp), Botucatu, SP, Brasil; 2 Faculdade de Medicina de Marília Departamento de Medicina Unidade de Tireoide Marília SP Brasil Unidade de Tireoide, Disciplina de Endocrinologia e Metabolismo, Departamento de Medicina, Faculdade de Medicina de Marília, Marília, SP, Brasil; 3 Universidade Federal da Bahia Instituto de Saúde e Ciência Departamento de Biorregulação Salvador BA Brasil Departamento de Biorregulação, Instituto de Saúde e Ciência, Universidade Federal da Bahia, Salvador, BA, Brasil; 4 Pontifícia Universidade Católica de Campinas Hospital Universitário Endocrinologia e Metabolismo Campinas SP Brasil Endocrinologia e Metabolismo, Hospital Universitário da Pontifícia Universidade Católica de Campinas (PUC-Campinas), Campinas, SP, Brasil; 5 Hospital Universitário Clementino Fraga Filho Departamento de Medicina Serviço de Endocrinologia Rio de Janeiro Brasil Departamento de Medicina, Serviço de Endocrinologia, Hospital Universitário Clementino Fraga Filho, Rio de Janeiro, Brasil; 6 Universidade Federal do Paraná Departamento de Clínica Médica Unidade de Tireoide Curitiba PR Brasil Unidade de Tireoide, Serviço de Endocrinologia, Departamento de Clínica Médica, Universidade Federal do Paraná, Curitiba, PR, Brasil

**Keywords:** Diagnosis, hypothyroidism, treatment

## Abstract

Primary hypothyroidism is a common disorder in clinical practice. The management of most cases of hypothyroidism is usually straightforward, but the best approach in some special situations may raise questions among physicians. This position statement was prepared by experts from the Brazilian Society of Endocrinology and Metabolism to guide the management of three special situations, namely, hypothyroidism in the elderly, subclinical hypothyroidism in patients with heart disease, and difficult-to-control hypothyroidism. The authors prepared the present statement after conducting a search on the databases MEDLINE/PubMed, LILACS, and SciELO and selecting articles with the best evidence quality addressing the selected situations. The statement presents information about the current approach to patients in these special situations.

## INTRODUCTION

Primary hypothyroidism is a frequent disorder worldwide (1). According to the Brazilian Longitudinal Study of Adult Health (ELSA-Brasil), the yearly incidences of overt and subclinical hypothyroidism in Brazil are about 0.5% and 1%, respectively (2).

Even though the diagnosis and treatment of hypothyroidism are usually uncomplicated, many physicians are unclear about the best approach in some special situations. Hypothyroidism in the elderly, subclinical hypothyroidism in patients with heart disease, and difficult-to-control hypothyroidism stand out among these special situations due to their common occurrence and potential clinical repercussions.

The incidence of hypothyroidism increases with age (2). This observation is particularly important considering the progressively increasing number and proportion of elderly individuals worldwide and a projected elderly population of about 1.4 billion individuals in 2030 (3). As age increases, so does the incidence of cardiovascular diseases. In Brazil, the reported rate of cardiovascular disease is around 687.5 cases per 100,000 inhabitants (4). Thus, considering the general risks and, particularly, the cardiovascular implications of hypothyroidism overtreatment and undertreatment, it is paramount for patients with hypothyroidism to be properly diagnosed and treated.

Regarding difficult-to-control hypothyroidism, this position statement focuses on refractory hypothyroidism. Even though the prevalence of refractory hypothyroidism is difficult to estimate, the frequency of patients with serum thyroid-stimulating hormone (TSH) levels outside the recommended goal during levothyroxine (LT4) treatment is elevated. Indeed, a Brazilian study has reported that around 28% of the patients treated with thyroid hormone replacement have elevated serum TSH levels (5). Questions regarding other forms of treatment for hypothyroidism arise in this situation, and interest in combined LT4 and liothyronine (LT3) therapy has increased over the last years.

Considering the concerns presented above, a group of experts from the Brazilian Society of Endocrinology and Metabolism (SBEM) prepared this position statement to guide diagnostic and therapeutic approaches to hypothyroidism in the three special situations mentioned above, *i.e.*, hypothyroidism in elderly individuals, subclinical hypothyroidism in patients with heart disease, and difficult-to-control hypothyroidism.

## MATERIALS AND METHODS

The Thyroid Department at SBEM selected two organizers for this position statement, who then invited collaborators with recognized expertise and relevant publications on the selected topics. Online discussion cycles were then held involving the collaborators and members of the Thyroid Department at SBEM, resulting in the elaboration of the three questions addressed in the present position statement:

- What is the approach to hypothyroidism in the elderly?
- When should subclinical hypothyroidism be treated in patients with heart disease?
- What is the approach to patients with difficult-to-control hypothyroidism?

The collaborators searched the databases MEDLINE/PubMed, LILACS, and SciELO for relevant publications on hypothyroidism. The keywords used in the search were hypothyroidism AND elderly AND diagnosis, hypothyroidism AND elderly AND treatment, and hypothyroidism AND heart disease AND treatment, difficult-to-control hypothyroidism OR refractory hypothyroidism AND diagnosis, difficult-to-control hypothyroidism OR refractory hypothyroidism AND treatment. The authors gave priority to articles with the best evidence quality (randomized trials and case-control, cohort, and cross-sectional studies), discussed the proposed questions, and prepared specific recommendations for each special situation, which are presented in the sections below.

## WHAT IS THE APPROACH TO HYPOTHYROIDISM IN THE ELDERLY?

### 1. How should the diagnosis of hypothyroidism be established in the elderly?

Overt hypothyroidism is characterized by increased TSH levels and decreased free thyroxine (FT4) levels, while subclinical hypothyroidism is characterized by increased TSH levels but normal thyroid hormone levels (6). Since circulating TSH increases with age, even in the absence of thyroid disease (7), age-specific TSH values must be considered, particularly in elderly individuals (Table 1) and among those with suspected subclinical hypothyroidism.

**Table 1:** Distribution by age group of the mean and 2.5th and 97.5th percentiles of serum thyroid-stimulating hormone (TSH) values obtained from 13,296 patients of all races and ethnicities and without thyroid disease

Age (years)	TSH (mIU/L)		
	2.5th percentile	Mean	97.5th percentile
13 to 19	0.41	1.30	3.78
20 to 29	0.40	1.30	3.60
30 to 39	0.38	1.25	3.60
40 to 49	0.44	1.40	3.90
50 to 59	0.49	1.50	4.20
60 to 69	0.46	1.66	4.70
70 to 79	0.47	1.74	5.60
> 80	0.44	1.90	6.30

Adapted from reference (8).

Normal serum TSH values vary according to age, sex, and ethnicity. Table 1 shows the results of the distribution of the mean and the 2.5th and 97.5th percentiles of TSH values among 13,296 North American individuals without thyroid disease (8). In a Brazilian study including 1200 subjects aged 20-80 years, the median upper TSH levels were 4.3 mIU/L, 5.8 mIU/L, and 6.7 mIU/L among individuals aged 20-59, 60-79, and > 80 years, respectively (9).

In patients with elevated serum TSH level, measurement of this hormone should be repeated within 3 months to confirm persistent dysfunction, along with measurement of serum FT4 to determine whether overt or subclinical hypothyroidism is present (6). In most circumstances, measurement of serum total triiodothyronine (T3) is not recommended. Of note, serum TSH levels may be mildly elevated during the recovery phase of nonthyroidal illnesses (6); therefore, patients with mildly elevated TSH levels who have been recently ill or hospitalized should have TSH levels rechecked in 6-8 weeks.

Once the diagnosis of thyroid dysfunction is confirmed, thyroid peroxidase antibody (TPOAb), a marker of autoimmunity, should be measured to investigate the occurrence of autoimmune thyroiditis. Elderly patients, in particular, may present abnormal laboratory tests associated with hypothyroidism; therefore, the workup in these patients should include, before treatment decision, a complete blood count, serum levels of sodium, total cholesterol, and low-density lipoprotein cholesterol, blood glucose values, and markers of nonalcoholic fatty liver disease (10,11).

#### Recommendation

- Overt hypothyroidism is characterized by increased TSH levels and decreased FT4 levels. The diagnosis of subclinical hypothyroidism in the elderly should be determined based on age-specific TSH values and after a repeated TSH measurement. Autoantibodies (TPOAb) should be measured in case of confirmed hypothyroidism or subclinical hypothyroidism.

### 2. When should overt hypothyroidism be treated in the elderly?

There is scant evidence on the harmful effects of overt hypothyroidism and the benefits of its treatment in elderly patients, as most studies are focused on the effects and treatment of subclinical hypothyroidism. Thus, recommendations about the treatment of these patients are based on the risks of not treating them and the historical experience acquired with thyroid hormone replacement over time.

Elderly patients with overt hypothyroidism require a careful and individualized clinical evaluation, since their symptoms – which are usually mild and unspecific – may be mistaken for those of other morbidities or attributed to the aging process (12,13). The most serious and life-threatening clinical presentation of hypothyroidism is myxedema coma, a condition that more frequently affects older individuals, exposing them to a greater risk of death (14). Heart failure is found in most cases of myxedema, usually with a fatal outcome if thyroid hormone replacement is not initiated in a timely manner (15). Patients with symptomatic heart failure have a higher risk of death when presenting with concomitant hypothyroidism compared with those with normal thyroid function (16). Severe hypothyroidism has also been associated with hypercholesterolemia, atherosclerosis, and coronary artery disease (17), while a recent systematic review and meta-analysis has found that LT4 replacement in overt hypothyroidism is associated with a significant improvement in lipid profile (18).

A meta-analysis of 27 cohort studies including 1,114,638 participants has found that overt hypothyroidism is significantly associated with increased all-cause mortality among the elderly (19). In another meta-analysis of 55 cohort studies including 1,898,314 participants, overt hypothyroidism was significantly associated with higher risks of ischemic heart disease, myocardial infarction, cardiac mortality, and all-cause mortality (20). In a Taiwanese population-based, retrospective cohort study including 2029 patients aged ≥ 65 years, hypothyroidism was independently associated with increased all-cause mortality, while LT4 replacement was associated with a lower risk of all-cause mortality (21). In another individual patient data (IPD) meta-analysis of four prospective studies including community-dwelling individuals aged ≥ 80 years, overt hypothyroidism was not associated with functional outcomes or mortality (22).

#### Recommendation

- Based on the risks of not treating elderly patients with hypothyroidism and the historical experience acquired with thyroid hormone replacement in these individuals, all elderly patients with overt hypothyroidism should be treated for symptom relief and prevention of clinical complications and/or unfavorable outcomes.

### 3. When should subclinical hypothyroidism be treated in the elderly?

Treatment has been recommended for younger (<65 years of age) and older (≥65 years of age) adults with subclinical hypothyroidism and TSH levels ≥ 10 mIU/L due to the association of this condition with increased cardiovascular and mortality risk (6,23–25). The most consistent evidence in this regard comes from an IPD meta-analysis (26) of 11 prospective cohort studies in which the risks of coronary heart disease (CHD) events and related mortality were significantly increased in participants with TSH levels ≥ 10 mIU/L, irrespective of age. In another IPD meta-analysis, the risk of heart failure also increased in patients with TSH levels ≥ 10.0 mIU/L, but not among oldest-old participants (≥80 years) (27). In a prospective study including older individuals (aged 70-82 years) with high cardiovascular risk, subclinical hypothyroidism with TSH level > 10 mIU/L was associated with an increased risk of incident heart failure (28). Conversely, other meta-analyses have suggested that these risks may only be present in younger but not in older individuals with subclinical hypothyroidism (29–32).

The clinical outcomes and mortality of subclinical hypothyroidism have also been investigated in studies including selected elderly populations (19,22). A systematic review found no association between subclinical hypothyroidism and all-cause or cardiovascular mortality among participants aged > 65 years (19). Another meta-analysis of participants aged ≥ 80 years has found no association between subclinical hypothyroidism and functional outcomes or mortality (22).

Few well-designed randomized controlled trials have been published on the effects of LT4 treatment in elderly individuals with subclinical hypothyroidism. The most important of these trials to date is the TRUST study (Thyroid Hormone Therapy for Older Adults with Subclinical Hypothyroidism), which involved 737 adults aged ≥ 65 years who had persistent subclinical hypothyroidism (33). In the TRUST study, LT4 therapy provided no apparent benefits on Hypothyroid Symptoms and Tiredness scores or on secondary outcomes. A lack of benefits from LT4 treatment was also reported in a secondary analysis of the TRUST study that included only participants with greater symptom burden at baseline (33,34). The impact of LT4 therapy has also been investigated in nested studies within the TRUST study, and no beneficial effects have been observed on carotid intima-media thickness (35), systolic and diastolic heart function (36), bone health (37), physical or mental fatigability (38), and depressive symptoms (39). Additionally, a Brazilian randomized controlled trial has found no relevant benefit on health-related quality of life of 6 months of LT4 therapy in women with subclinical hypothyroidism (40). A prospectively planned analysis of data from two clinical trials including adults aged ≥ 80 years with subclinical hypothyroidism has found that treatment with LT4, compared with placebo, was not significantly associated with improvement in hypothyroid symptoms or fatigue (41). Also, in pooled data from two parallel randomized controlled trials (42) – TRUST and IEMO80+ (the Institute for Evidence-Based Medicine in Old Age 80-plus thyroid trial) – treatment with LT4 did not significantly change the risk of all cardiovascular outcomes in older adults with subclinical hypothyroidism, irrespective of history of cardiovascular disease or age. Finally, a meta-analysis of randomized controlled trials including 2192 participants with subclinical hypothyroidism (43) has shown that LT4 treatment was not associated with improvements in general quality of life or thyroid-related symptoms.

These data combined show no clear evidence of unfavorable clinical outcomes from subclinical hypothyroidism or any benefit of treatment with LT4 among older adults (particularly oldest-old individuals) with subclinical hypothyroidism. In contrast, LT4 overtreatment in elderly individuals has been consistently associated with a higher risk of adverse effects, such as atrial fibrillation (44), osteoporosis, and bone fractures (45). Notably, a Brazilian multicenter study has found that almost 15% of the patients with hypothyroidism use LT4 at supraphysiological doses (5).

#### Recommendation

- The decision to treat elderly patients with subclinical hypothyroidism should be based on the patient's individual clinical characteristics and on a careful balance between treatment risks and benefits.
- Treatment should be considered for older patients (≥65 years) with TSH levels persistently above 10 mIU/L, particularly those with high cardiovascular risk. However, a more cautious and conservative approach is recommended for oldest-old patients (≥ 80 years), particularly those older than 85 years, in whom the treatment risks can outweigh the potential benefits. A wait-and-see approach including regular monitoring of thyroid function, considering quality of life and life expectancy, seems to be the best strategy for these patients.

### 4. How should the treatment of hypothyroidism be in the elderly?

The drug of choice for thyroid replacement in the elderly is LT4 because of its proven clinical efficacy and safety profile (23,24,46). There is no recommendation for combined LT4/LT3 therapy in elderly patients due to lack of evidence of benefit with this approach (47) and higher risk of adverse effects associated with overtreatment, such as atrial fibrillation (44) and osteoporotic fractures (45).

There is scant evidence on the most appropriate LT4 dose to initiate treatment of hypothyroidism in the elderly. The initial dose varies depending on the patient's clinical context, including the hypothyroidism severity, presence of comorbidities, and use of medications. Healthy older adults without cardiac disease, particularly those aged < 80 years, can be safely treated with a full starting dose of LT4 (48). On the other hand, in the oldest old (aged > 80 years) and in patients with severe comorbidities (particularly coronary artery disease and heart failure), a low starting dose (12.5-25 μg) followed by progressive dose titration every 4-8 weeks is a good strategy to avoid overtreatment or precipitation of ischemic heart disease (48,49).

Compared with younger individuals, older individuals usually require a lower total LT4 dose due to lower lean body mass and decreased metabolism and thyroxine turnover (49,50). Still, several other factors seem to influence the LT4 dose in the elderly, including the presence of comorbidities, use of medications, decreased LT4 absorption, and low adherence. Thus, the total LT4 requirement varies in older individuals (51), and the frequency of excessive or insufficient LT4 replacement in this group of patients is relatively high (5,52).

The optimal target TSH level in elderly patients on LT4 treatment is unclear, but since these patients are more vulnerable to the adverse effects of overtreatment, establishing as a target value a TSH level between 2.0-6.0 mIU/L is safe in preventing both excessive and insufficient LT4 treatment (53).

#### Recommendation

- The drug of choice for thyroid hormone replacement therapy in the elderly is LT4, while combined LT4/LT3 therapy should be avoided in this population.
- Healthy older adults aged < 80 years may be started on a full-dose replacement regimen, while for oldest-old individuals (particularly those with underlying comorbidities), a partial replacement regimen with lower starting doses (12.5-25 μg) is a safer approach.
- The LT4 dose should be adjusted every 4–8 weeks considering as a target value a TSH level between 2.0 and 6.0 mIU/L.

## WHEN SHOULD SUBCLINICAL HYPOTHYROIDISM BE TREATED IN PATIENTS WITH HEART DISEASE?

Thyroid hormones exert marked effects on the heart, and both overt and subclinical hypothyroidism have been associated with an increased risk of cardiovascular morbidity and mortality (26,20,32). However, no well-powered and well-designed study has been conducted to assess whether treating subclinical hypothyroidism is beneficial in terms of cardiovascular risk. Thus, the decision to treat a patient with subclinical hypothyroidism and heart disease depends on a balance between the risks of not treating and the potential benefits of treating the patient.

Subclinical hypothyroidism has been consistently associated with increased levels of serum total cholesterol, low-density lipoprotein cholesterol, and triglycerides (18,54,55). Endothelial dysfunction has also been associated with subclinical hypothyroidism (56,57), but the effect of this condition on blood pressure remains unclear (58). In a meta-analysis of six cohort studies, the risk of heart failure events increased with high TSH levels, particularly when ≥ 10 mIU/L (27). In another meta-analysis, subclinical hypothyroidism, compared with normal thyroid function, was associated with an increased risk of all-cause mortality and cardiac death and/or hospitalization in patients with heart failure with reduced ejection fraction (59). A meta-analysis including 55,287 participants from 11 prospective cohorts has shown an association between subclinical hypothyroidism and higher risk of CHD events and CHD mortality in individuals with TSH levels ≥ 10 mIU/L (26). Notably, the risk of CHD mortality was significantly increased at serum TSH levels ≥ 7.0 mIU/L (26). Also, in two large-scale meta-analyses, subclinical hypothyroidism was associated with an increased risk of cardiovascular disease and all-cause mortality, especially at TSH levels ≥ 10 mIU/L (20,32) and among individuals with high cardiovascular risk (32), although consistent evidence has suggested that these risks are limited to patients younger than 65 years (29,32). Indeed, studies including participants from selected elderly populations found no association between subclinical hypothyroidism and all-cause and cardiovascular mortality (19,22).

The benefits of treating subclinical hypothyroidism in patients with heart disease have not been evaluated in well-powered, randomized controlled studies. However, growing body of evidence (18,54,57,60–64) has shown favorable effects of LT4 treatment on atherogenic lipid profile and carotid intima-media thickness in patients with subclinical hypothyroidism. The treatment of subclinical hypothyroidism on cardiovascular risk and mortality has been associated with conflicting results in longitudinal and retrospective cohort studies (65–68). A prospective, well-designed study found no beneficial effect of LT4 treatment on left ventricular function in patients with subclinical hypothyroidism and acute myocardial infarction (69). In contrast, a systematic review and meta-analysis of observational and randomized studies has found that LT4 replacement was significantly associated with a lower all-cause and cardiovascular mortality in younger (<65-70 years) but not older (≥65-70 years) adults (70).

In conclusion, despite evidence of detrimental cardiovascular risks associated with subclinical hypothyroidism, there is lack of high-quality studies on the benefits of treating subclinical hypothyroidism to reduce these risks. Thus, the decision of whether to treat or not a patient with subclinical hypothyroidism and heart disease is increasingly complex and should be based on the individual characteristics of each patient.

### Recommendations

- Younger patients (<65 years of age) with heart disease or at high cardiovascular risk presenting with subclinical hypothyroidism should be treated when serum TSH levels are ≥ 10 mIU/L to avoid the risks of cardiac failure, endothelial dysfunction, CHD events, and death.
- Younger patients (<65 years of age) with heart disease or at high cardiovascular risk presenting with subclinical hypothyroidism with TSH > 7.0 mIU/L should be considered for treatment to avoid the risk of CHD mortality.
- Treatment with LT4 should be considered in patients with subclinical hypothyroidism and atherogenic dyslipidemia, particularly when TSH is ≥ 10 mU/mL.

## WHAT IS THE APPROACH TO PATIENTS WITH DIFFICULT-TO-CONTROL HYPOTHYROIDISM?

In terms of difficult-to-control hypothyroidism, an important aspect is the approach to patients with refractory hypothyroidism, when potential causes and therapeutic alternatives for this condition should be further explored. Another concern in difficult-to-control hypothyroidism is the management of patients who remain symptomatic despite presenting with serum thyroid hormone levels within the recommended goals, raising considerations about the benefits of combined LT4/LT3 therapy in this situation.

### 1. How should the approach be to refractory hypothyroidism?

Refractory hypothyroidism is a term used for patients with primary hypothyroidism who require daily doses of LT4 above 2.5 μg/kg or 250-300 μg (71). Persistently high serum TSH levels must be investigated to identify whether the patient is taking LT4 correctly and to exclude factors affecting LT4 bioavailability. In these conditions, the investigation should include the patient's compliance and possible interference with LT4 bioavailability by nutrients, foods, drugs, and gastrointestinal diseases (Table 2). Potential malabsorption syndromes affecting LT4 bioavailability include (i) atrophic gastritis due to the presence of parietal cell antibodies, (ii) atrophic gastritis associated with *Helicobacter pylori* and hypochloridria, and (iii) reduced LT4 absorption related to celiac disease, among other causes (72).

**Table 2:** Conditions associated with persistently elevated serum thyroid-stimulating hormone (TSH) levels during treatment with levothyroxine (LT4)*

Insufficient LT4 dose for body weight
Non-compliance or incorrect LT4 administration
Inappropriate tablet storage
TSH heterophile antibodies
Addison's disease
Use of drugs that affect LT4 requirement Drugs that should not be co-administered with LT4: calcium (citrate, carbonate, or acetate), ferrous sulfate, antacids, proton pump inhibitors, simethicone, orlistat, bile acid sequestrants Drugs that can potentially change LT4 requirements: estrogens, raloxifene, phenobarbital, tricyclic antidepressants, sertraline, beta-blockers, rifampin, ciprofloxacin, tyrosine kinase inhibitors, amiodarone, and lithium
Conditions that interfere with LT4 absorption Celiac disease (gluten enteropathy), inflammatory bowel disease, lactose intolerance, pernicious anemia, pangastritis, *Helicobacter pylori* infection, motility problems, parasitic diseases (*e.g*., giardiasis), small intestine bacterial overgrowth, short bowel syndrome, gastric banding, sleeve gastrectomy, and Roux-en-Y gastric bypass

* Modified from references (92–94).

Lack of adherence to LT4 treatment is common and should be explored with the patient in a non-confrontational manner. The oral LT4 absorption test may be used to discriminate between gastrointestinal malabsorption versus pseudomalabsortion, the latter caused by an intentional lack of treatment adherence by the patient. The test is performed by administering to the patient a fixed oral LT4 dose of 1.0-1.5 mg (1,000-1,500 μg) and measuring serum TSH levels along with free T4 (FT4) or total T4 (TT4) at baseline and at 1-hour intervals for 4 to 6 hours. Gastrointestinal malabsorption is excluded in the presence of a TT4 elevation > 50% or a twofold to threefold increase in FT4, peaking at 2 hours, and a TSH decrease by 40% after 2 hours (73–75).

#### Recommendation

- In hypothyroidism refractory to treatment with LT4, it is recommended to:
  – verify compliance and correct administration of LT4 (with water, on an empty stomach, and at least 30 minutes before breakfast or at bedtime);
  – reassess whether the LT4 dose is adequate for the patient's body weight;
  – ensure that LT4 is not being taken with food, beverages, coffee, milk, papaya, fibers, or soy-containing products;
  – investigate potential drugs affecting LT4 requirement;
  – exclude medical conditions that interfere with LT4 absorption;
  – consider the oral LT4 absorption test to discriminate between gastrointestinal malabsorption versus pseudomalabsortion;
  – avoid combined therapy with LT4/LT3, as this approach is not routinely recommended.

### 2. What are the therapeutic alternatives to treat hypothyroidism?

Many studies have shown that up to 40% of the patients with hypothyroidism are overtreated and up to 40% are undertreated, especially when older than 65 years (52,76). A Brazilian study including 2057 patients with hypothyroidism has shown prevalence rates of undertreatment and overtreatment of 25.9% and 14.4%, respectively, and 80% of failure by the patients to follow the physicians' instructions due to forgetfulness or prescription misunderstandings (77). Patients' noncompliance can occur due to limitations and inconveniences imposed by LT4 intake, including the requirements of waiting approximately 30 minutes for the next meal, taking the medication daily and during fasting, and giving at least 1 hour before taking other medications. Many attempts have been made to improve patients' compliance to LT4 therapy, and new therapeutic alternatives have been suggested (72).

The influence of food intake on LT4 absorption was first reported by Wenzel & Kirschsieper, who demonstrated that LT4 absorption was significantly better when LT4 was taken during fasting (79%) than with simultaneous food intake (64%) (78). Other recent studies have also evaluated the effect on serum TSH levels of the timing of LT4 administration in relation to food intake. Levels of TSH have been shown to be higher when LT4 is taken with breakfast versus during fasting, although the levels remained within the normal range in both conditions (79,80). Bolk and cols. evaluated the effects of switching LT4 intake from morning to bedtime. Compared with LT4 taken in the morning, the bedtime intake was associated with a mean decrease in TSH level of 1.25 mIU/L and mean increases in FT4 and T3 levels of 0.07 ng/dL and 6.5 ng/dL, respectively (81). The administration of LT4 concomitant to breakfast or at bedtime, as opposed to fasting, could be more convenient for patients, and studies have shown that despite a mild elevation in TSH levels, administration of LT4 in these alternative timings maintained the TSH levels within the normal range (80,81).

A randomized, crossover study with LT4 administered once daily versus once weekly has found that once weekly LT4 treatment leads to transient increases in FT4 levels compared with daily LT4 treatment (1.91 ± 0.49 ng/dL versus 1.13 ± 0.25 ng/dL, respectively) but is not associated with hyperthyroidism, cardiac symptoms, echocardiographic abnormalities, or significant differences in mean TSH levels between groups (3.45 ± 2.67 mIU/L versus 1.87 ± 1.60 mIU/L, respectively) at 6 weeks (82). Once weekly LT4 administration was safe and well-tolerated and was not associated with evidence of treatment toxicity, particularly cardiac effects (82,83).

Patients who are unable to take LT4 while fasting may be prescribed LT4 in gel capsules or liquid (84–86), which – unlike LT4 tablets – are not dependent on gastric pH for proper absorption. Gel capsules deliver LT4 dissolved in glycerin and supplied in gelatin capsules, while the liquid formulation delivers sodium LT4 dissolved in glycerin or ethanol, also supplied in gelatin capsules. These alternative formulations can be taken with breakfast without affecting TSH levels.

Intramuscular LT4 has been used to treat refractory hypothyroidism in patients with an abnormal LT4 absorption test, in whom TSH levels normalized on a regimen of intramuscular 500 μg LT4 once or twice per week (87,88).

#### Recommendation

- Alternative LT4 formulations may be an option for patients with difficult treatment adherence and, rarely, in patients with a negative LT4 absorption test. For patients in whom a specific serum TSH goal is important (e.g., pregnant women and patients who require TSH suppression for cancer treatment), LT4 tablets during fasting are the recommended treatment.

### 3. Should patients be treated with levothyroxine-liothyronine combination?

While most patients respond well to LT4 therapy, some – for unknown reasons – remain symptomatic even when presenting with TSH levels within the normal range, imposing another treatment challenge. The persistence of symptoms of hypothyroidism despite apparently normal laboratory tests could be due to the several causes shown in Table 3. Excluding these causes, it is unclear why some patients remain symptomatic despite taking an optimal LT4 dose, and the role of combined LT4/LT3 therapy in this circumstance is questionable (89).

**Table 3:** Conditions associated with hypothyroidism-like symptoms that may be present in patients with persistent symptoms despite adequate levothyroxine treatment and normal thyroid-stimulating hormone (TSH) levels*

Anemia
Vitamin B12 deficiency
Folate deficiency
Vitamin D deficiency
Addison's disease
Hypopituitarism
Celiac disease
Hyperparathyroidism
Fatigue due to other causes (*e.g.,* lifestyle factors, depression, fibromyalgia, chronic fatigue syndrome, obesity, obstructive sleep apnea, sleep disturbances, alcohol, drug abuse)

* Medication adherence, symptoms, and changes in medical circumstances must always be verified. Modified from references (92–94).

The rationale for administering LT4 to patients with hypothyroidism lies in the peripheral conversion of T4 to the metabolically active T3 by the action of two enzymes, deiodinase type 1 (D1) and deiodinase type 2 (D2). With the acceptance of serum TSH measurement as the primary test to manage patients with hypothyroidism, and T3 identified as the biologically active thyroid hormone, a valid question that has emerged has been whether therapy with LT4 alone can restore serum T3 levels to the normal range (89). While the reasons why some patients fail to present complete resolution of symptoms remain unclear, a polymorphism (Thr92Ala) in the deiodinase 2 gene (*DIO2*) that encodes D2 (which converts T4 to T3 in the brain) has been identified more often in patients who have persistent symptoms while receiving LT4 monotherapy and may predict a better response to combination LT4/LT3 therapy (90). However, identification of these patients before initiating LT4 therapy is not currently feasible, and genetic testing for the Thr92Ala D2 polymorphism is not recommended.

The guidelines on the treatment of hypothyroidism by the American Thyroid Association (ATA) recognize that patients with hypothyroidism treated with LT4 and with normal serum TSH values may have serum T3 levels at the lower end of – or even below – the reference range (46). The significance of T3 concentrations within or slightly below the normal range is still unclear, and no sufficient evidence exists to recommend LT4 treatment with the goal of achieving normal/low values of TSH or normal/high values of T3 (46).

Evidence from experimental models of hypothyroidism has shown that LT4 alone may be unable to deliver an adequate amount of T3 to all tissues targeted by thyroid hormone action, while this can be achieved by combined LT4/LT3 therapy (73). Several clinical trials have been unable to show consistent benefits with combined LT4/LT3 therapy, and the benefits of this approach must still be demonstrated (91). Important aspects of combined LT4/LT3 administration must be clarified before this therapy is recommended to a patient (Table 4). Recent scientific developments may provide insight into the inconsistent results observed with LT4/LT3 therapy and guide future studies (92).

**Table 4:** Important considerations about treatment with combined levothyroxine-liothyronine (LT4/LT3)

Lack of evidence showing advantage of combination LT4/LT3 over LT4 alone for routine use. Larger and double-blinded, well-designed trials are still needed to evaluate different dosing regimens (T4 to T3 proportion), longer treatment durations, ability of the combined treatment in providing physiologic thyroid hormone levels, and validated outcomes.
LT3 and thyroid extract therapy are not commercially available in Brazil.
Controlled trials have shown lack of additional benefits of combined LT4/LT3 therapy over LT4 monotherapy in terms of quality of life, mood, or psychometric measures.
Some individuals prefer combined LT4/LT3 therapy, but long-term safety data supporting the routine use of this therapy are limited.
A trial of combined LT4/LT3 therapy may be considered in carefully selected patients following an informed discussion about the potential adverse consequences of excessive LT4 replacement. Combined LT4/LT3 must be avoided in pediatric patients, pregnant women, and elderly individuals.
The impact of type 2 deiodinase gene (*DIO2*) Thr92Ala polymorphism (rs225014) in patients undergoing LT4 therapy should be further investigated and can open up opportunities for a more personalized approach to hypothyroidism treatment.
Physicians have been prescribing LT3-containing therapies, but more research is required regarding patients’ preferences and benefits.

Modified from references (47,91,95–97).

#### Recommendations

- In the case of persistent symptoms attributable to hypothyroidism despite apparent normal laboratory tests, it is recommended to:
  – investigate nutritional, endocrine, and autoimmune conditions that might account for such symptoms;
  – explore the differential diagnosis of fatigue (workup plan suggestion: investigate lifestyle factors, depression, fibromyalgia, chronic fatigue syndrome, obesity, obstructive sleep apnea, sleep disturbances, alcohol, or drug abuse);
  – consider physiological changes that occur as adult patients progress through different stages of life and experience changes in LT4 needs related to medical conditions and medications;
  – contemplate referring the patient to an endocrinologist if symptoms persist;
  – avoid combined LT4/LT3 therapy, considering that until now there is lack of solid evidence showing advantage of LT4/LT3 over LT4 alone.

## FINAL CONSIDERATIONS

This position statement addresses the approach in three situations requiring special care in patients with hypothyroidism, *i.e.*, hypothyroidism in elderly patients, subclinical hypothyroidism in patients with heart disease, and difficult-to-control hypothyroidism. Even though the approach to overt hypothyroidism in the elderly is straightforward, the diagnosis of subclinical hypothyroidism in this population must take into consideration specific TSH thresholds for each age group, while treatment decisions must consider a balance between risks and benefits for each individual. Patients with difficult-to-control hypothyroidism should be initially investigated regarding treatment adherence, proper use of LT4, and conditions potentially interfering with LT4 absorption/metabolization. If symptoms suggestive of hypothyroidism persist despite normal thyroid hormone levels, other causes for the symptoms should be investigated.

In conclusion, the approach to primary hypothyroidism is usually uncomplicated and should consider the individual characteristics of each patient, while some special situations covered in this statement demand greater attention in terms of investigation and treatment.
